# Carotid plaque segmentation and classification using MRI-based plaque texture analysis and convolutional neural network

**DOI:** 10.3389/fmed.2025.1502830

**Published:** 2025-06-20

**Authors:** Zakarya Hasan Ahmed Abu Alregal, Gehad Abdullah Amran, Ali A. Al-Bakhrani, Saleh Abdul Amir Mohammad, Amerah Alabrah, Lubna Alkhalil, Abdalla Ibrahim, Maryam Ghaffar

**Affiliations:** ^1^School of Computer Science and Technology, Central South University, Changsha, China; ^2^Department of Management Science and Engineering, Dalian University of Technology, Dalian, China; ^3^College of Software Engineering, Dalian University of Technology, Dalian, China; ^4^STC's Artificial Intelligence Chair, Department of Information Systems, College of Computer and Information Sciences, King Saud University, Riyadh, Saudi Arabia; ^5^School of Automation, Central South University, Changsha, China; ^6^Department of Computer Engineering, Khwaja Fareed University of Engineering and Information Technology, Rahim Yar Khan, Pakistan; ^7^Department of Radiology, The Affiliated Changsha Central Hospital, Hengyang Medical School, University of South China, Changsha, China

**Keywords:** carotid plaque classification, MRI, stroke risk assessment, plaque texture analysis, deep learning, segmented plaques

## Abstract

**Background:**

Accurate segmentation and classification of carotid plaques are critical for assessing stroke risk. However, conventional methods are hindered by manual intervention, inter-observer variability, and poor generalizability across heterogeneous datasets, limiting their clinical utility.

**Methods:**

We propose a hybrid deep learning framework integrating Mask R-CNN for automated plaque segmentation with a dual-path classification pipeline. A dataset of 610 expert-annotated MRI scans from Xiangya Hospital was processed using Plaque Texture Analysis Software (PTAS) for ground truth labels. Mask R-CNN was fine-tuned with multi-task loss to address class imbalance, while a custom 13-layer CNN and Inception V3 were employed for classification, leveraging handcrafted texture features and deep hierarchical patterns. The custom CNN was evaluated via K10 cross-validation, and model performance was quantified using Dice Similarity Coefficient (DSC), Intersection over Union (IoU), accuracy, and ROC-AUC.

**Results:**

The Mask R-CNN achieved a mean DSC/IoU of 0.34, demonstrating robust segmentation despite anatomical complexity. The custom CNN attained 86.17% classification accuracy and an ROC-AUC of 0.86 (*p* = 0.0001), outperforming Inception V3 (84.21% accuracy). Both models significantly surpassed conventional methods in plaque characterization, with the custom CNN showing superior discriminative power for high-risk plaques.

**Conclusion:**

This study establishes a fully automated, hybrid framework that synergizes segmentation and classification to advance stroke risk stratification. By reducing manual dependency and inter-observer variability, our approach enhances reproducibility and generalizability across diverse clinical datasets. The statistically significant ROC-AUC and high accuracy underscore its potential as an AI-driven diagnostic tool, paving the way for standardized, data-driven cerebrovascular disease management.

## 1 Introduction

**Figure 1:**
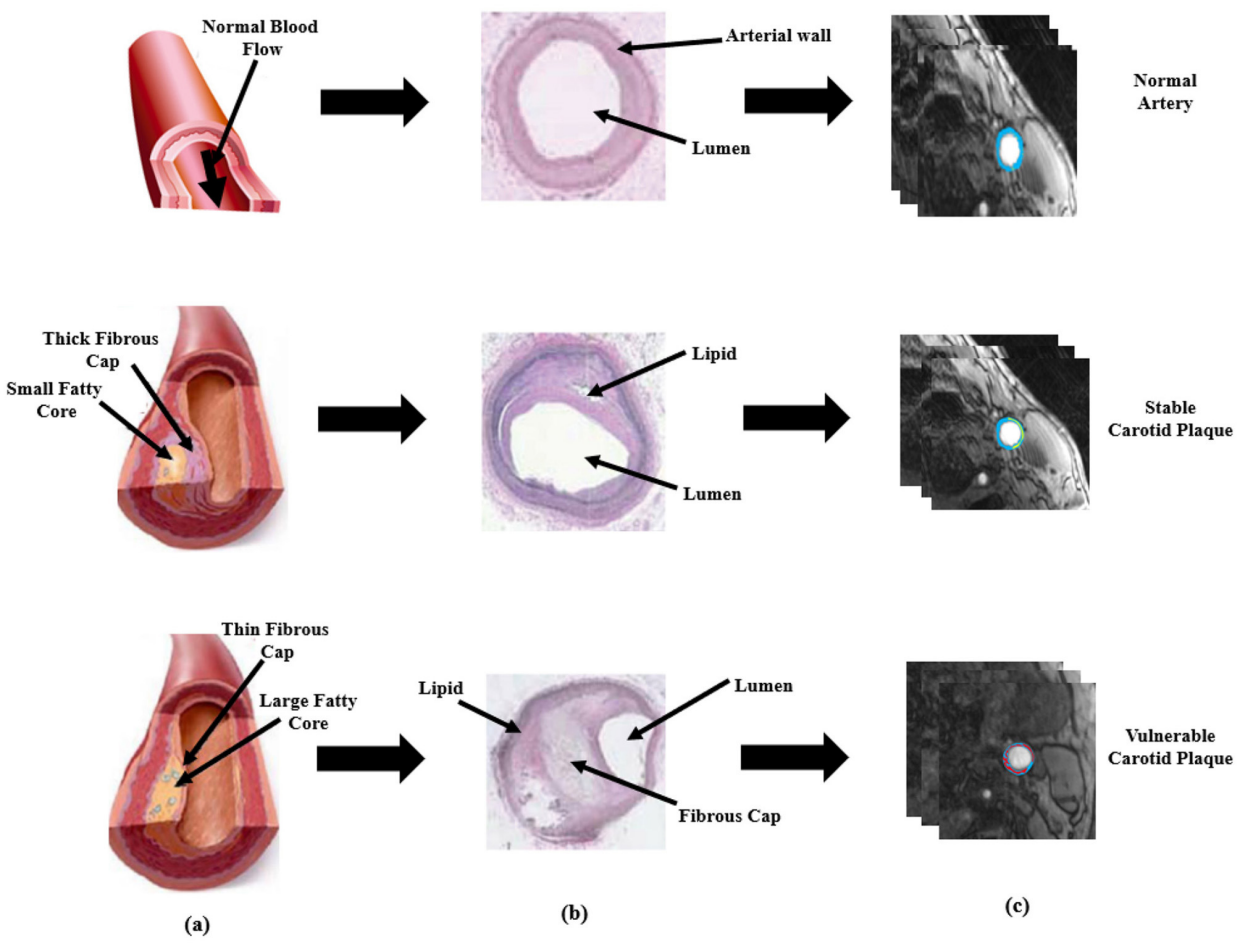
Visual representation of plaque formation, histological representation and PTAS segmentation. **(a)** is the schematic description based on normal healthy artery, the diseased artery with stable plaque, and with vulnerable plaque **(b)** is the histological representation of normal stable, and vulnerable artery and **(c)** is plaque segmentation using PTAS.

Each year, 647,000 Americans die from cardiovascular diseases (CVD), which account for 17.9 million deaths worldwide. Benjamin et al. (1), which means a loss of one life in every 37s (2). The main causes of CVD are the development of atherosclerosis and the production of plaque in the vasculature, including the coronary and carotid arteries (3). A thrombus is frequently formed as a result of plaque rupture or plaque ulceration, which can embolize or occlude the lumen, restricting blood flow and resulting in myocardial infarction or stroke (4). As the plaque falls off, the components such as platelets and cellulose formed in the blood will adhere and accumulate, which in turn creates a thrombus and causes obstruction of the distal artery. This lead to less blood to the brain, causing cerebral ischemia and hypoxia disease, that is, bloody stroke often referred to as cerebral infarction and increase the risk of stroke (5). Figure 1 gives a visual comparison between a normal artery and an artery with plaque buildup along with the histological representation and their respective plaque segmentation using PTAS.

Public health is seriously affected by stroke. The percentage of the vessel that is stenotic due to the lumen being obstructed by atherosclerotic plaque is currently used as one of the clinical reasons for carotid endarterectomy (CAE) or carotid stenting (CAS). Patients with recent cerebrovascular symptoms and high-grade carotid stenosis experience a 17% absolute risk reduction for stroke over a two-year period in large CEA trials, such as the North American Symptomatic Carotid Endarterectomy Trial. As a result, patients can be treated with both preventive and therapeutic treatments through early planning, detection, and monitoring of high-risk populations, such as those with cardiovascular and cerebrovascular disorders. Since the total plaque area is likewise susceptible to changes in the plaque, doctors can detect changes in the plaque based on the area of the plaque. Medical practitioners frequently do carotid plaque segmentation manually, albeit this takes time, depends on the operator's clinical background, and may result in incorrect diagnoses that are risky for the patient's life. Magnetic resonance imaging (MRI) remains the preferred technique for evaluating and monitoring plaques due to its widespread application in clinical settings (6), alongside other imaging methods such as computed tomography (CT) (7) and ultrasound (US) (8). MRI's ability to deliver superior soft tissue contrast compared to CT and US, combined with the potential to integrate multiple contrast-weighted sequences, provides enhanced diagnostic information (9). Furthermore, MRI offers key advantages, including its operator-independence and the absence of ionizing radiation, making it a safer option for repeated use. Despite these benefits, there is a notable gap in comprehensive research on MRI-based radiomics specifically targeting carotid plaque, with current literature limited to exploratory analyses, such as MRI texture characterization of basilar artery plaques. The computer-aided framework was used in this study to identify and categorize carotid artery atherosclerotic plaques. Machines may be better able to manage linear and non-linear fluctuations in plaque distribution because of the significant and fuzzy pixels data obtained from the spatial magnetic resonance imaging; the recent trend in artificial intelligence (AI) has been used to characterize and classify plaques (10) using machine learning (ML). Deep learning technology has recently been used in various applications of life, including image classification (11), and medical imaging (12). In particular, radiological imaging has been can be better analyzed by deep learning (DL) technology (13). The neural network layers of the DL architecture dynamically adapt the fluctuations in grayscale contrast and automatically produce down-sampled representations of the original pattern (14). In the domain of multiple sclerosis lesion segmentation, Kim et al. (15) employed a 3-layer convolutional encoder network to generate segmentation predictions that matched the resolution of the original images. In a different study (16), a segmentation technique utilizing the Hough transform was proposed for carotid magnetic resonance imaging; however, the method proved unsuitable for carotid plaque segmentation due to the artery's curved structure. Loizou et al. (17) developed an advanced segmentation framework based on the snake model for carotid artery images, though its reliance on manual initialization of the snake can result in inaccurate outcomes. Another methodology, introduced by Destrempes et al. (18), used a combination of three Nakagami distributions to model the echogenicity in carotid magnetic resonance images. This approach simulated plaques, vascular lumens, and external arterial walls but required manual segmentation of the initial image frame, limiting full automation.

Here are the key contributions of this study, highlighting significant advancements in the classification and segmentation of carotid plaques for the assessment of stroke risk. The following points detail the innovative methods and results achieved through this research.

- Curated and processed 610 high-resolution MRI scans from Xiangya Hospital, ensuring robust model training and validation, with expert-annotated ground truth labels enhancing clinical applicability.
- Adapted and fine-tuned Mask R-CNN specifically for MRI-based carotid plaque segmentation, incorporating a multi-task loss function and Dice similarity coefficient to mitigate class imbalance and improve segmentation robustness.
- Developed a dedicated 13-layer CNN tailored for medical imaging, effectively capturing intricate plaque characteristics, while integrating Inception V3 to leverage deep hierarchical features, achieving state-of-the-art classification accuracy (86.17% with CNN, 84.21% with Inception V3).
- Designed a fully automated and scalable framework that reduces dependency on manual assessment, minimizes inter-observer variability, and enhances stroke risk assessment, paving the way for AI-assisted diagnostic support in cerebrovascular disease management.

This study bridges critical gaps in carotid plaque analysis by introducing a fully automated, hybrid deep learning framework that synergizes advanced segmentation with multi-scale classification to optimize stroke risk stratification. Integrating Mask R-CNN's instance-aware segmentation capabilities–trained on expert-annotated MRI scans and optimized via multi-task loss–with a purpose-built 13-layer CNN and Inception V3, our approach addresses the limitations of manual intervention and inconsistent feature extraction inherent to conventional methods. Using automating feature learning while reducing inter-observer variability, this work establishes a scalable paradigm for AI-driven cerebrovascular diagnostics, offering a pathway toward standardized, data-driven stroke risk assessment in diverse clinical populations.

## 2 Related work

The analysis of carotid plaques is a critical component of stroke risk assessment, yet significant challenges remain in achieving accurate and automated segmentation and classification. Over the years, various methodologies have been proposed to address these challenges, ranging from traditional image processing techniques to advanced deep learning models. This section provides an in-depth review of the state-of-the-art approaches, emphasizing recent advancements and identifying gaps that this study aims to address.

### 2.1 Early approaches: image processing and classical machine learning

Early efforts in carotid plaque analysis relied on conventional image processing techniques and classical machine learning algorithms. Methods such as thresholding, edge detection, and region-growing were commonly used for plaque segmentation (16). These techniques, while straightforward, are highly sensitive to variations in imaging conditions and often fail to capture the irregular shapes and fuzzy boundaries of plaques. Another limitation is their reliance on manual tuning of parameters, which makes them impractical for large-scale applications.

Classical machine learning approaches, such as Support Vector Machines (SVM) and Random Forests, have also been explored. These methods typically involve extracting handcrafted features, such as texture, intensity, and shape, and using them to train classifiers. For instance, Destrempes et al. (18) modeled echogenicity using Nakagami distributions to segment plaques, lumens, and arterial walls. However, this approach required manual initialization, limiting its scalability. Similarly, Loizou et al. (17) developed a snake model-based framework for carotid artery segmentation, but its performance was heavily dependent on operator expertise. While these methods demonstrated some success, they are inherently limited by their inability to generalize across diverse datasets and complex plaque morphologies.

### 2.2 Transition to deep learning: convolutional neural networks (CNNs)

The advent of deep learning has revolutionized medical imaging, enabling automated feature extraction and significantly improving performance. Convolutional Neural Networks (CNNs) have become the cornerstone of many state-of-the-art solutions for medical image analysis. Architectures like U-Net (19) and nnU-Net (20) have been widely adopted for segmentation tasks due to their ability to capture fine-grained details in medical images. These models have achieved remarkable success in segmenting tumors, lesions, and other anatomical structures, demonstrating their potential for carotid plaque analysis.

Recent studies have also explored the use of Mask R-CNN (21) for medical image segmentation. Unlike traditional CNNs, Mask R-CNN extends Faster R-CNN by adding a branch for pixel-level segmentation, making it suitable for tasks requiring both object detection and precise boundary delineation. Although Mask R-CNN has been applied to various medical imaging tasks, its application to carotid plaque segmentation remains underexplored. Additionally, pre-trained architectures like Inception V3 (22) and ResNet (23) have been fine-tuned for classification tasks, leveraging transfer learning to achieve high accuracy with limited data. These advancements highlight the growing potential of deep learning in medical imaging but also underscore the need for specialized frameworks tailored to carotid plaque analysis.

### 2.3 Recent advances: radiomics and multi-modality integration

In recent years, radiomics has emerged as a promising approach for extracting quantitative features from medical images. By analyzing texture, shape, and intensity patterns, radiomics-based methods provide deeper insights into disease characteristics and progression. For example, Khanna et al. (13) demonstrated the utility of radiomics in characterizing rheumatoid arthritis using MRI sequences. While radiomics has shown promise in oncology and neurology, its application to carotid plaque analysis is still in its infancy.

Another emerging trend is the integration of multi-modality data, such as combining MRI with ultrasound or CT scans, to improve diagnostic accuracy. Recent work by Liu et al. (6) explored the use of multi-modal imaging for carotid plaque characterization, achieving better performance than single-modality approaches. However, these methods often require complex preprocessing and alignment, making them computationally expensive and challenging to implement in clinical settings.

Despite the significant advancements in medical imaging and deep learning, several critical gaps and challenges persist in the domain of carotid plaque analysis, which this study directly addresses. First, most existing research focuses on other vascular regions, such as the basilar artery, or unrelated diseases like multiple sclerosis, leaving carotid plaque segmentation underexplored. This lack of attention to carotid plaques has created a pressing need for specialized solutions tailored to stroke risk assessment, which our study fulfills by leveraging a robust dataset of 610 MRI scans from Xiangya Hospital specifically annotated for carotid plaques. Second, existing models often struggle with the irregular shapes and fuzzy boundaries of carotid plaques, leading to suboptimal segmentation accuracy. To address this, we fine-tuned Mask R-CNN with a multi-task loss function and Dice similarity coefficient, achieving a mean DSC of 0.34 and laying the groundwork for further improvements. Third, few studies integrate segmentation and classification into a unified pipeline, resulting in fragmented workflows that hinder clinical adoption. Our hybrid framework bridges this gap by combining Mask R-CNN for precise segmentation with a custom CNN and Inception V3 for classification, streamlining the diagnostic process and enhancing clinical applicability. Finally, many traditional methods rely on manual initialization or parameter tuning, limiting their scalability and practicality in real-world settings. By designing a fully automated and scalable framework, our study minimizes human intervention, reduces inter-observer variability, and paves the way for AI-assisted diagnostic support in cerebrovascular disease management. Through these contributions, our work not only addresses the limitations of existing approaches but also sets a new benchmark for carotid plaque analysis.

## 3 Materials and methods

**Figure 2:**
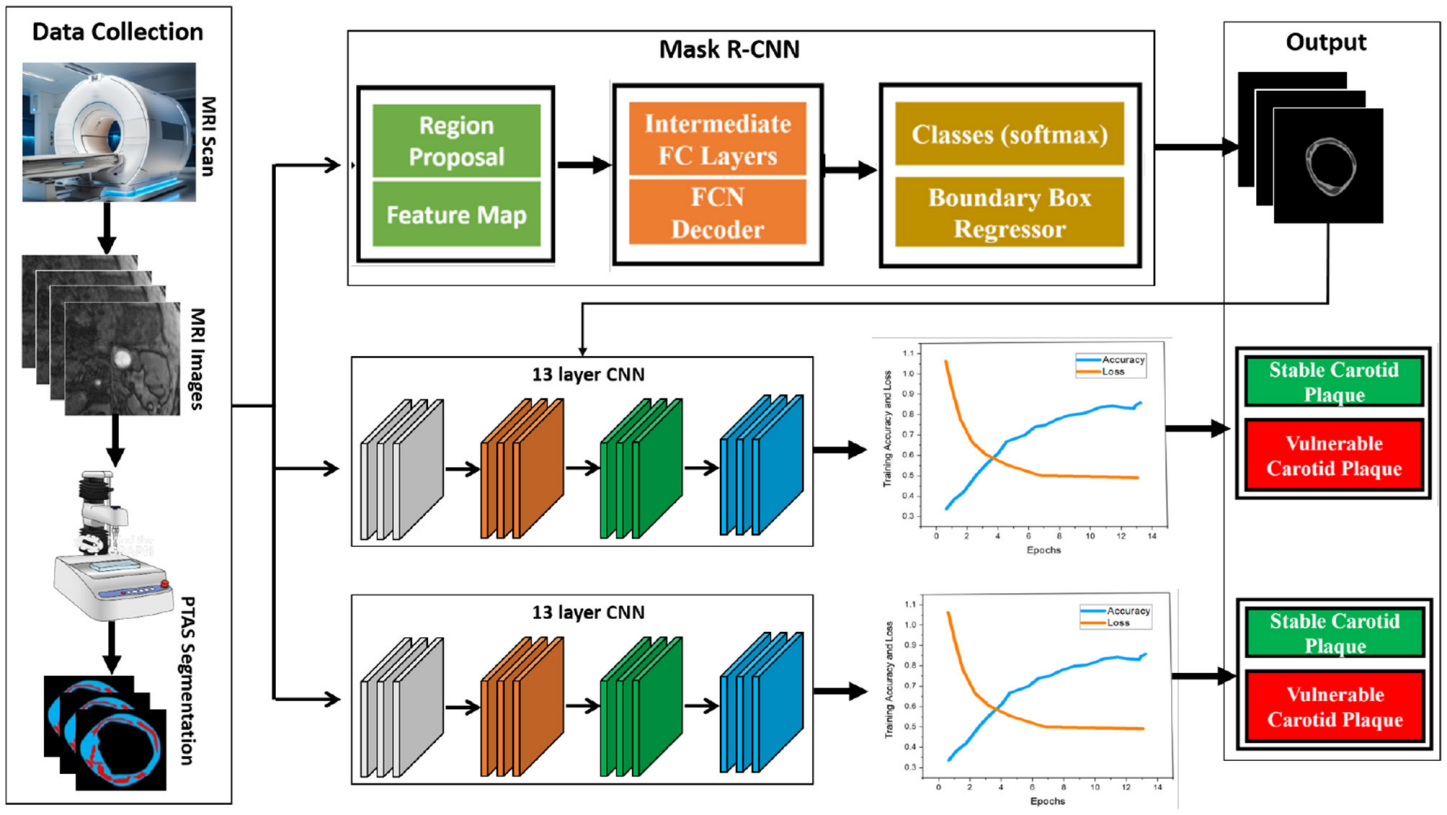
Proposed framework for this study. First, raw magnetic resonance images are processed using the Plaque Texture Analysis to trace the Region-of-Interest (ROI) and trained Mask R-CNN for automatic plaque segmentation; then, these data are the input for the 13-layered convolution neural network and Inception V3 for training and classification.

In this study, our proposed model consists of two parts: the use of Plaque Texture Analysis Software (PTAS) for the manual plaque segmentation protocol to trace the region of interest and then the Mask R-CNN to perform automatic carotid plaque segmentation. While in the second part, we used a 13-layer traditional convolution neural network and Inception V3 for the training and classification of stable and vulnerable carotid plaque for stroke risk assessment. Our proposed model is shown in Figure 2.

### 3.1 Data collection and statistics

**Figure 3:**
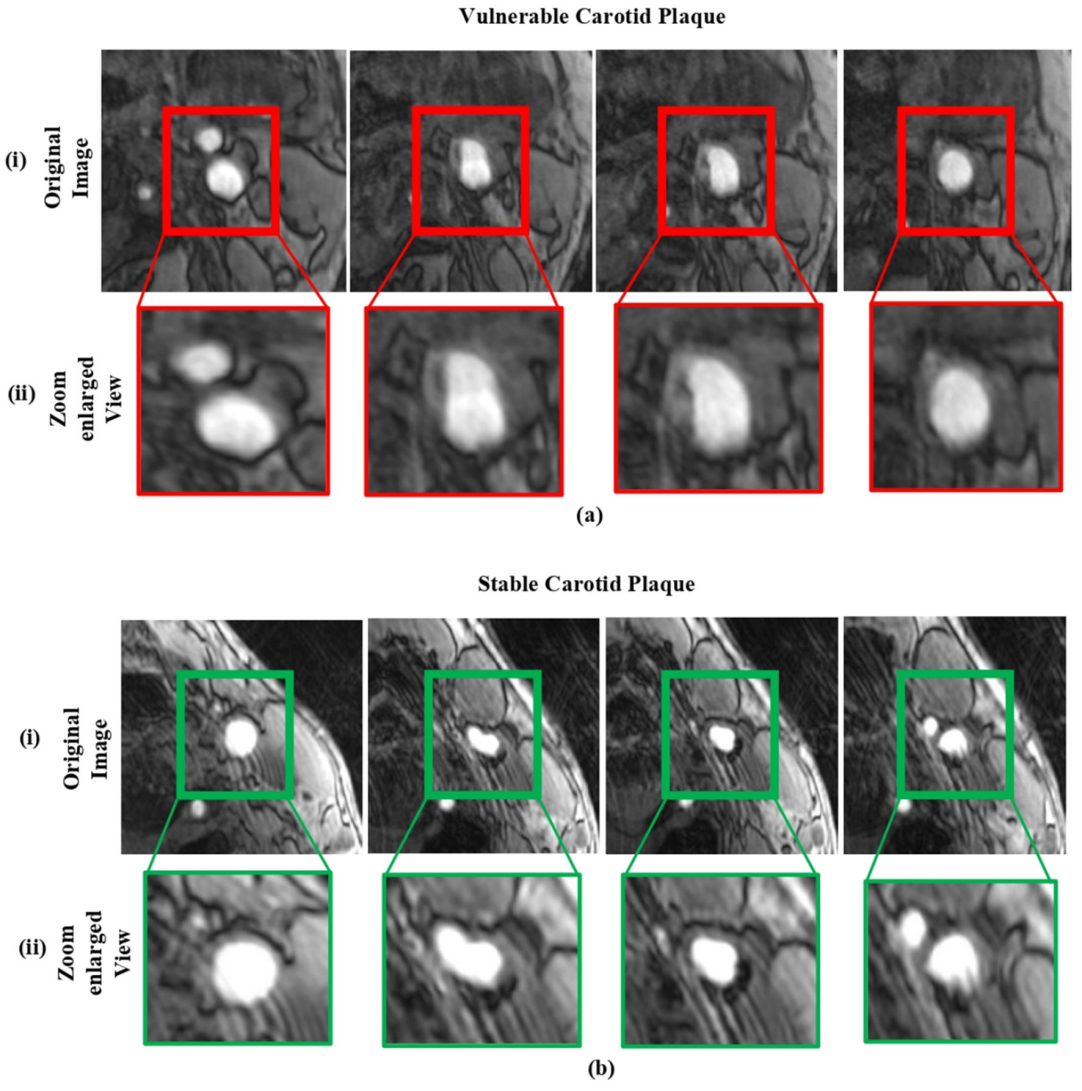
The sample dataset: **(a)** includes magnetic resonance images of vulnerable carotid plaques, and **(b)** includes images of stable carotid plaques. In each case, (i) shows the original magnetic resonance images, while (ii) displays their corresponding enlarged views.

The FDA/CFDA-approved magnetic resonance examination technology named the magnetic resonance plaque analysis system (MRI-VPD) is used in this study. The data included normal patients, and susceptible cases were collected from the Xiangya Hospital of Central South University. We selected the 106 hospital patients who had risk indicators for atherosclerosis. Given the inherent complexity of medical images and the stringent accuracy requirements for their analysis, such tasks are typically carried out by expert professionals. In this study, we randomly selected a total of 610 sample images. To ensure an effective evaluation, the dataset was partitioned into 90% for training and 10% for testing. A detailed representation of the sample data is provided in Figure 3.

The dataset used in this study consists of MRI images from 106 patients diagnosed with risk indicators for atherosclerosis. These images represent both vulnerable and stable carotid plaques, and the data was partitioned into 90% for training and 10% for testing. The demographic details of the dataset, including patient age, gender, ethnicity, and risk factors, were carefully considered to ensure a diverse and representative sample. Several measures, including random selection, stratified sampling, and balancing demographic variables, were employed to mitigate potential biases and improve the generalizability of the model. The full details of the dataset are shown in Table 1.

**Table 1:** Summary of the dataset used in this study, including details on patient demographics and bias mitigation strategies.

**Category**	**Description**
Total number of patients	106 patients
Total number of images	610 sample images
Image categories	Vulnerable carotid plaques, stable carotid plaques
Training set	90% of images (549 images)
Testing set	10% of images (61 images)
Age range	18–30, 31–50, 51–70, >70
Gender	Male, Female
Ethnicity	Han, Minority ethnic groups (e.g., Miao, Tujia)
Risk factors	Hypertension, hyperlipidemia, diabetes, family history
Inclusion criteria	Patients with atherosclerosis risk factors (hypertension, hyperlipidemia, diabetes, family history)
Exclusion criteria	Patients with interventions or conditions that could interfere with plaque morphology interpretation
Bias mitigation	Random selection, stratified sampling by plaque type, and consideration of demographic diversity

#### 3.1.1 Inclusion criteria

Identifying carotid artery stenosis is crucial, as it can be detected through various imaging techniques such as ultrasound, computed tomography angiography (CTA), magnetic resonance angiography (MRA), and other numerical simulations (24), even in the absence of clinical symptoms. Unlike some other imaging tests, which may miss significant stenosis or only identify transient ischemic attacks (TIA) and cerebral infarctions of uncertain origin, magnetic resonance imaging of the carotid arteries offers detailed insights.

#### 3.1.2 Scanning parameters

A Philips 3.0T MRI scanner with an 8-channel phased array surface coil, optimized for carotid artery imaging, was employed. Patients were asked to lie on the scanning table, stay still, avoid swallowing, and ensure their jaw and neck were centered in the coil. The imaging protocol began with coronal thin slice T2-weighted imaging (T2WI) to capture both carotid arteries. These images were then processed to visualize the arterial structure and identify any areas of stenosis. Detailed information on the imaging sequence and parameters is provided below:

- Rapid gradient echo (3D MERGE): this sequence utilized a 3D motion-sensitized driven equilibrium preparation with fast gradient echo (turbo field echo). The repetition time (TR) and echo time (TE) were set at 9 ms and 4.2 ms, respectively. The field of view (FOV) was 25 × 16 × 4 cm3, with a spatial resolution (SR) of 0.8 × 0.8 × 0.8 mm3 and a flip angle of 6°. The imaging duration was 4 minutes.
- 3D simultaneous non-contrast angiography and intra-plaque hemorrhage (3D SNAP): this sequence employed turbo field echo with a TR of 10 ms and TE of 4.8 ms. The FOV was 25 × 16 × 4 cm3, spatial resolution was 0.8 × 0.8 × 0.8 mm3, and the flip angles were 11° and 5°. The total imaging time was 5 minutes.
- 3D Time of Flight (TOF): Utilized fast field echo with TR and TE of 20 ms and 4.9 ms, respectively. The FOV was 16 × 16 × 4 cm3, with a spatial resolution of 0.6 × 0.6 × 2 mm3 and a flip angle of 20°. Imaging lasted 6 minutes. Axial 3D TOF, along with fast spin echo (FSE)-based T1-weighted imaging (T1WI) and T2-weighted imaging (T2WI), were conducted in the longitudinal range of 20–24 mm (10–12 slices), with the stenosis centered and fat suppression applied. Consistent positioning was maintained for T1WI, T2WI, and 3D TOF sequences. Images of patients with carotid plaques were selected for further analysis. Post-processing was carried out using the MRI-VPD system, and Plaque View software was used to analyze the properties and components of carotid plaques. All analysis and measurement steps were independently performed by three senior radiologists. Informed consent was obtained from the patients and their families.

### 3.2 Plaque segmentation using PTAS

The carotid plaque is classified into two classes, vulnerable and stable carotid plaque, for the purpose of assessing the risk of stroke. The plaque segmentation methodology tries to manually trace the region of interest in the anterior and posterior walls of the carotid artery. We used “Plaque Texture Analysis Software (PTAS)” (Iconsoft International Ltd, Greenford, London, UK) to trace and segment the magnetic resonance images, just like in earlier studies (25, 26). It has two advantages: first, you can normalize the images so that the adventitia layer's median gray-level intensity is between 180 and 190 (bright intensities), and blood's median gray-level intensity is between 0 and 5. Second, the PTAS delineation approach is user-friendly (25). This implies that after normalization, the physician can use a mouse to create a brand-new image of the plaque's outline and preserve it. The calcified plaques cast acoustic shadows, but they weren't taken into account when defining their boundaries. This made it possible to choose the plaque's calcification- and non-calcification-related elements outside of the acoustic shadow. The complete process is carried out and validated by the domain experts. We trained the Mask R-CNN network to automatically segment plaque in magnetic resonance images following the manual segmentation of the pictures.

### 3.3 Plaque segmentation using Mask R-CNN

Image segmentation, which is the process of splitting an image into numerous segments or regions that correspond to various items or sections of an object, is a common technique that uses Mask R-CNN. The input for Mask R-CNN is an enhanced image multiplied by the edge map acquired via HED in order to grasp the structure. This mask identifies the pixels in the image that belong to the object, allowing for fine-grained segmentation of the object. We utilize a current Tensor Flow deep learning implementation that is open-source (26). We use an efficient method like Adam Optimizer (27) to solve this problem and improve training. The multi-task loss function of the Mask R-CNN, as given in Equation 1, combines the classification, localization, and segmentation mask losses.


(1) $$\begin{array}{l} L=L_{\text{cls}}+L_{\text{box}}+L_{\text{mask}} \end{array}$$

**Figure 4:**
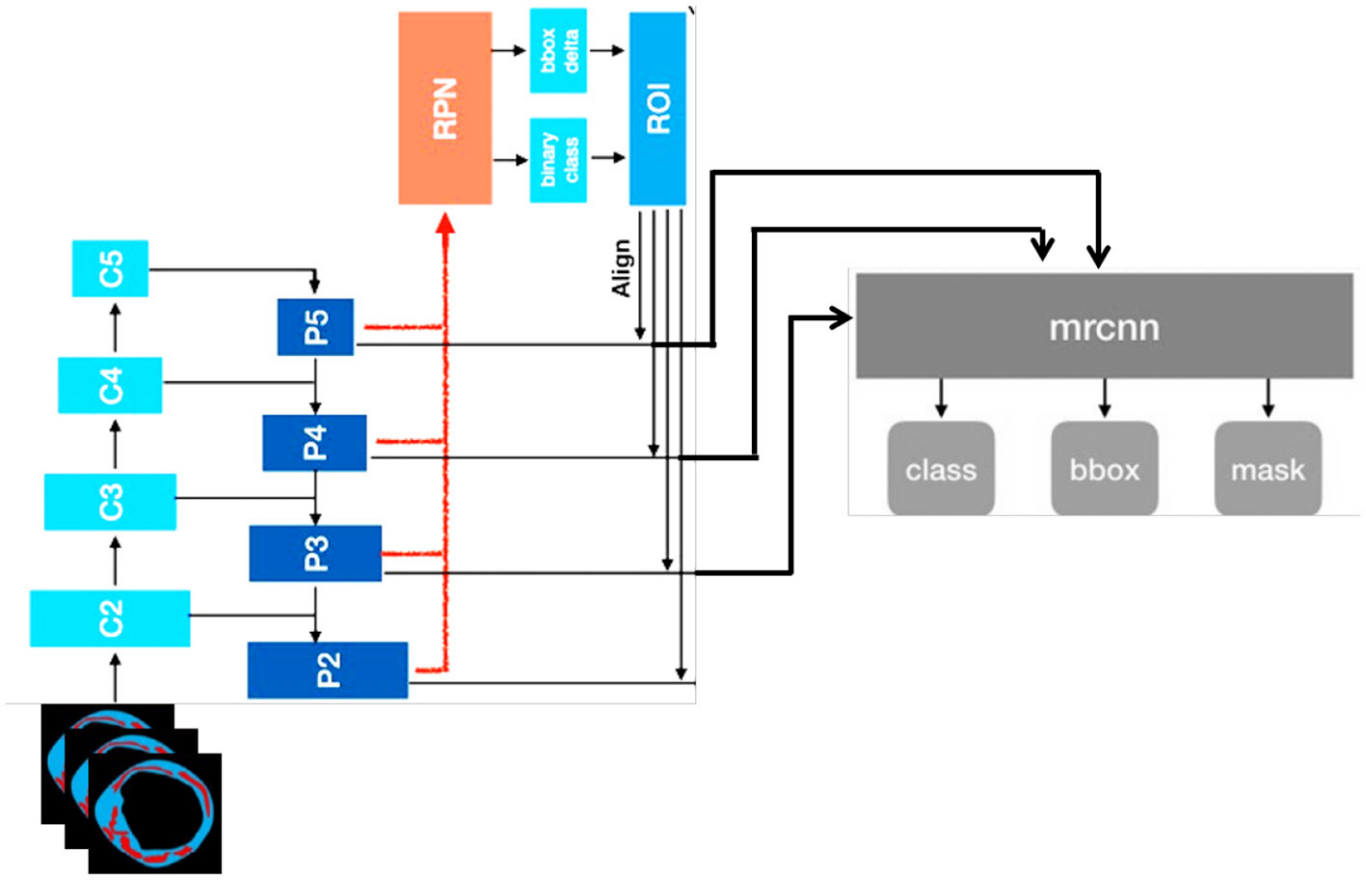
Architecture of mask R-CNN used in this study.

*L*_box_ is the bounding box regression loss, *L*_mask_ is the mask loss, and *L*_cls_ is the classification loss. To solve the class-imbalance issues, the Dice coefficient was used. We substituted the Dice coefficient loss for the binary cross-entropy loss in the *L*_mask_ loss modification. We observed that the validation loss and Dice coefficient loss converged smoothly. The complete structure of Mask R-CNN is shown in Figure 4.

### 3.4 Deep learning architecture

**Figure 5:**
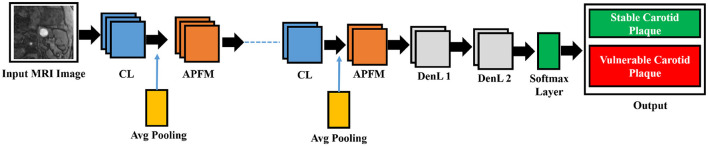
The five CL, five APL, two DenL, and one fattened layer comprise the conceptual view of the deep learning architecture. The missing three CL and three APL layers are represented by the dashed line in the middle.

Size and image size are equally reasonable and range from 55 × 43 to 593 × 107 pixels due to our cohort. The 13-layered CNN design we chose has four convolution layers (CL), four average pooling layers (APL), two dense layers, and one dropout layer, as illustrated in Figure 5.

We optimized the model by modifying hyperparameters including layer types, dropout rates, momentum, and learning rates. The softmax layer, which is the final layer of the network, computes the categorical cross-entropy loss function (E) between the symptomatic and asymptomatic patient groups, as described in Equation 2.


(2) $$\begin{array}{l} E=-\left[y*\log\left(p\right)+\left(1-y\right)*\log\left(1-p\right)\right] \end{array}$$

In Equation 2, *y* is the binary indicator for the observed class, and *p* is the predicted probability of the plaque belonging to a specific class, calculated using deep learning. The product is denoted by *. Equation 3 shows the number of output features from the average pooling feature mappings (APFM), whereas Equation 4 shows the number of output features from the convolution procedure (28).


(3) $$\begin{array}{l} n_{\text{out}}=\left\lfloor \frac{n_{\text{in}}+\left(2*p\right)-M}{S}\right\rfloor +1 \end{array}$$

(4) $$\begin{array}{l} a_{\text{out}}=\left(\frac{w-f}{s}\right)+1 \end{array}$$

The symbols used in Equations 3, 4 represent key parameters in the convolutional and pooling operations. The variables *n*_in_ and *n*_out_ denote the number of input and output feature maps, respectively. The parameter *w* corresponds to the width of the input feature map, while *f* represents the kernel size used in convolution operations. The convolution kernel size is denoted by *M*, and the padding size is given by *P*. The stride, represented by *S*, determines the step size of the kernel movement during the convolution process. These parameters collectively influence the feature extraction process and define the spatial dimensions of the transformed feature maps in deep learning models.

In each convolutional layer (CL), *n*_in_ and *n*_out_ specify the numbers of input and output features, respectively. Table 2 details the layers' names, feature map sizes, and training parameters in three distinct columns. The parameters include *n*_out_ (number of output features), *w* (width of the input feature map), *f* (kernel size), *M* (convolution kernel size), *P* (convolution padding size), and *S* (stride size, which indicates kernel movement).

**Table 2:** Details of hyperparameters used in the CNN Model for our dataset.

**Sn**	**Name of the layer**	**C2 feature map size**	**C3 trainable parameter**
R1	2D convolution layer-1	(240, 240, 256)	7,168
R2	2D average pooling layer-1	(120, 120, 256)	0
R3	2D convolution layer-2	(120, 120, 128)	295,040
R4	2D average pooling layer-2	(60, 60, 128)	0
R5	2D convolution layer-3	(60, 60, 64)	73,792
R6	2D average pooling layer-3	(30, 30, 64)	0
R7	2D convolution layer-4	(30, 30, 32)	18,464
R8	2D average pooling layer-4	(15, 15, 32)	0
R9	2D convolution layer-5	(15, 15, 16)	4,624
R10	2D average pooling layer-5	(7, 7, 16)	0
R11	1D flatten	784	0
R12	1D dense-1	128	100,480
R13	Dropout layer	128	0
R14	1D Dense-2	2	258
R15	Total trainable parameters		499,826

**Figure 6:**
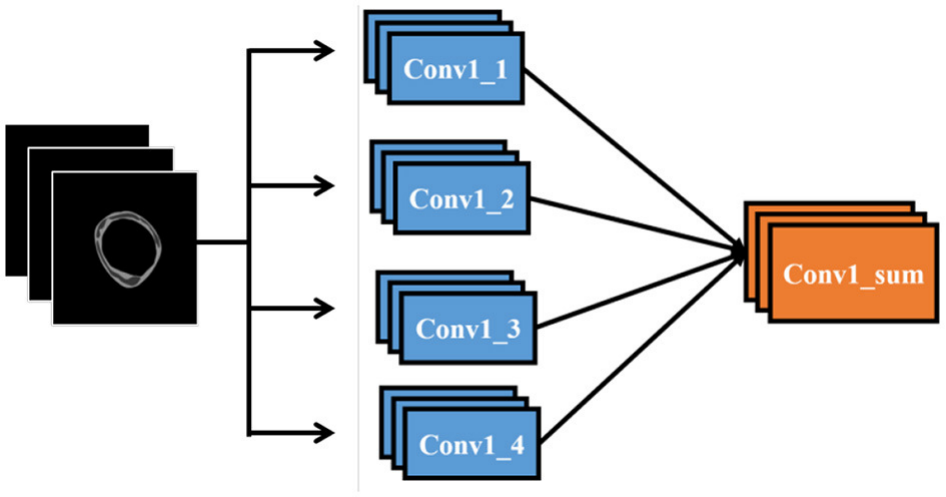
Modified CNN Input Layers in our proposed architecture.

To integrate our data into a CNN model, we replicate the first convolutional layer four times and connect each copy to the respective data layers. These four convolutional layers are then combined through a sum-up layer, which merges multiple feature maps of identical size into a single feature map. This combined feature map is subsequently linked to the remainder of the network. Figure 6 illustrates the modifications made to the input section of the network

### 3.5 Inception V3

**Figure 7:**
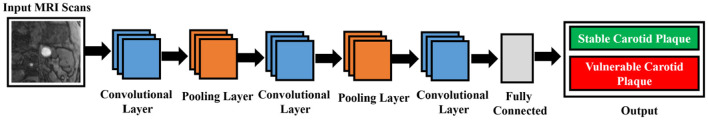
Schematic diagram of Inception V3 network structure.

The primary goal of Inception v3, which improves upon earlier designs, is to reduce computational resource usage (22). Inception Networks are more computationally efficient than VGG-16 networks in terms of the number of parameters and the computational effort required. This is achieved by decomposing larger two-dimensional convolutions into smaller one-dimensional convolutions. For example, a 7 × 7 convolution is split into a 1 × 7 convolution and a 7 × 1 convolution, and a 3 × 3 convolution is divided into a 1 × 3 convolution and a 3 × 1 convolution. This method significantly reduces the number of parameters, minimizes memory overhead, and enhances network performance. After filtering and denoising, the data is forwarded to the designated network for training. The network structure is illustrated in Figure 7.

Inception v3, as shown in Equation 5, is a deep convolutional neural network model with 48 layers, pre-trained on over one million images from the ImageNet dataset.

The symbols in Equation 5 represent key components of the matrix multiplication operation used in feature extraction. The matrix *A*_*X*_ represents the transformed feature map, while *A* and *B* denote input matrices of dimensions *M*×*N*. Each element *A*_*i, j*_ and *B*_*i, j*_ corresponds to specific feature values extracted from the input image. The summation terms iterate over the indices *i* and *j* to compute the final feature representation, capturing spatial dependencies in convolutional layers.


(5) $$\begin{array}{c} A_{X}=\left[\begin{array}{ccc} A_{1,1} & \cdots & A_{1,N}\\ A_{21} & \cdots & A_{2N}\\ A_{M1} & \cdots & A_{MN} \end{array}\right]*\left[\begin{array}{ccc} B_{1,1} & \cdots & B_{1N}\\ B_{21} & \cdots & B_{2N}\\ B_{M1} & \cdots & B_{MN} \end{array}\right]\\ =\overset{M-1}{\underset{i=0}{\sum }}\overset{N-1}{\underset{j=0}{\sum }}A_{\left(M-1\right),\left(N-j\right)}B_{\left(i+1\right),\left(j+1\right)} \end{array}$$

The complete model architecture and hyper parameter details are shown in Table 3.

**Table 3:** Hyper parameters details used in the Inception V3 model according to our dataset.

**Layer (type)**	**Output shape**	**Param**
inception_v3 (Model)	(None, 8, 8, 2,048)	21,802,784
flatten_1 (Flatten)	(None, 131,072)	0
activation_95 (Activation)	(None, 131,072)	0
dropout_1 (Dropout)	(None, 131,072)	0
dense_1 (Dense)	(None, 1024)	134,218,752
activation_96 (Activation)	(None, 1,024)	0
dropout_2 (Dropout)	(None, 1,024)	0
dense_2 (Dense)	(None, 28)	28,700
activation_97 (Activation)	(None, 2)	0

### 3.6 Evaluation metrics

The Receiver Operating Characteristic (ROC) curve (29) is utilized to compare the effectiveness of two or more diagnostic methods, providing a comprehensive assessment of a test's diagnostic accuracy. The ROC-AUC, also referred to as the Area Under the Curve (AUC), is explained in Equation 6.


(6) $$\begin{array}{c} P\left(x_{1}>x_{0}\right)=P\left(x_{1}-x_{0}>0\right) \end{array}$$

In this context, *x*_1_ and *x*_0_ are continuous random variables representing the “scores” assigned by our binary classifier to randomly selected positive and negative samples, respectively. The ROC curve shows the trade-off between sensitivity (True Positive Rate, TPR) and specificity (False Positive Rate, FPR), as given by Equations 7, 8. These metrics are essential for evaluating the classifier's performance in distinguishing between the two classes.


(7) $$\begin{array}{c} \text{TPR}=\frac{\text{TP}}{p} \end{array}$$

(8) $$\begin{array}{c} \text{FPR}=\frac{\text{FP}}{N} \end{array}$$

Based on the convolution theorem, with the assumption of convergence (30), the expression for ROC-AUC is formulated and presented in Equation 9.


(9) $$\begin{array}{c} P\left(x_{1}>x_{0}\right)=P\left(x_{1}>x_{0}\right)>0=\int_{0}^{+\infty }\int_{-\infty }^{+\infty }f_{1}\left(u\right)Xf_{0}\left(u-v\right)dudv \end{array}$$

Let *x*_1_ and *x*_0_ represent continuous random variables that correspond to the “scores” assigned by the binary classifier to randomly selected positive and negative samples, respectively.

Moreover, we also used a confusion matrix to evaluate our model's performance. Each column of the matrix represents a class instance prediction, while each row represents an actual class instance. It is so named because this matrix can analyze whether the machine has confused two classes (28). There is a list of rates that are often computed from a confusion matrix for a binary classifier which are described below (31): Equation 10 depiction of recall explains how many of the positive groups we properly predicted.


(10) $$\begin{array}{c} \text{Recall}=\frac{\text{TP}}{TP+FN} \end{array}$$

Equation 11 presents the formula for precision, which measures the accuracy of positive predictions made by the model. Precision is defined as the ratio of true positives to the total number of predicted positives.


(11) $$\begin{array}{c} \text{Precision}=\frac{\text{TP}}{TP+FP} \end{array}$$

Accuracy is a metric for evaluation of classification models which shows the fraction of predictions and the accuracy is calculated using the formula shown in Equation 12.


(12) $$\begin{array}{c} \text{Accuracy}=\frac{\text{TP}}{TP+TN} \end{array}$$

## 4 Experimental results

In this research work, we used PTAS to trace the ROI (the plaque in the carotid arteries) from the magnetic resonance images and then trained Mask R-CNN for automatic segmentation of plaque from magnetic resonance images. We used 13-layered CNN and Inception V3 for the image classification. The model was trained on a hardware setup featuring an Intel Core i7 processor and a Tesla K40 C graphics card. The training was conducted using a Python 3.6 environment with TensorFlow on an Ubuntu 16.04 LTS system. The learning rate was manually configured to 10^−3^, and the weights were initialized to zero.

### 4.1 Experimental results for plaque segmentation

To assess the model's performance in segmenting plaques in new images, we calculated four evaluation metrics: the Dice similarity score (DSC) of (0.736 ± 0.10), the intersection over union (IoU) of (0.583 ± 0.12), and Cohen's Kappa coefficient of (0.728 ± 0.10). The threshold that yielded the highest mean DSC and IoU values was found to be 0.34. The mean and standard deviation for each evaluation metric are detailed in Table 4.

**Table 4:** Evaluation metrics of the plaque segmentation.

**Evaluation metrics**	**Average (±std)**
DSC	0.736 ± 0.10
IoU	0.583 ± 0.12
KI	0.728 ± 0.10

**Figure 8:**
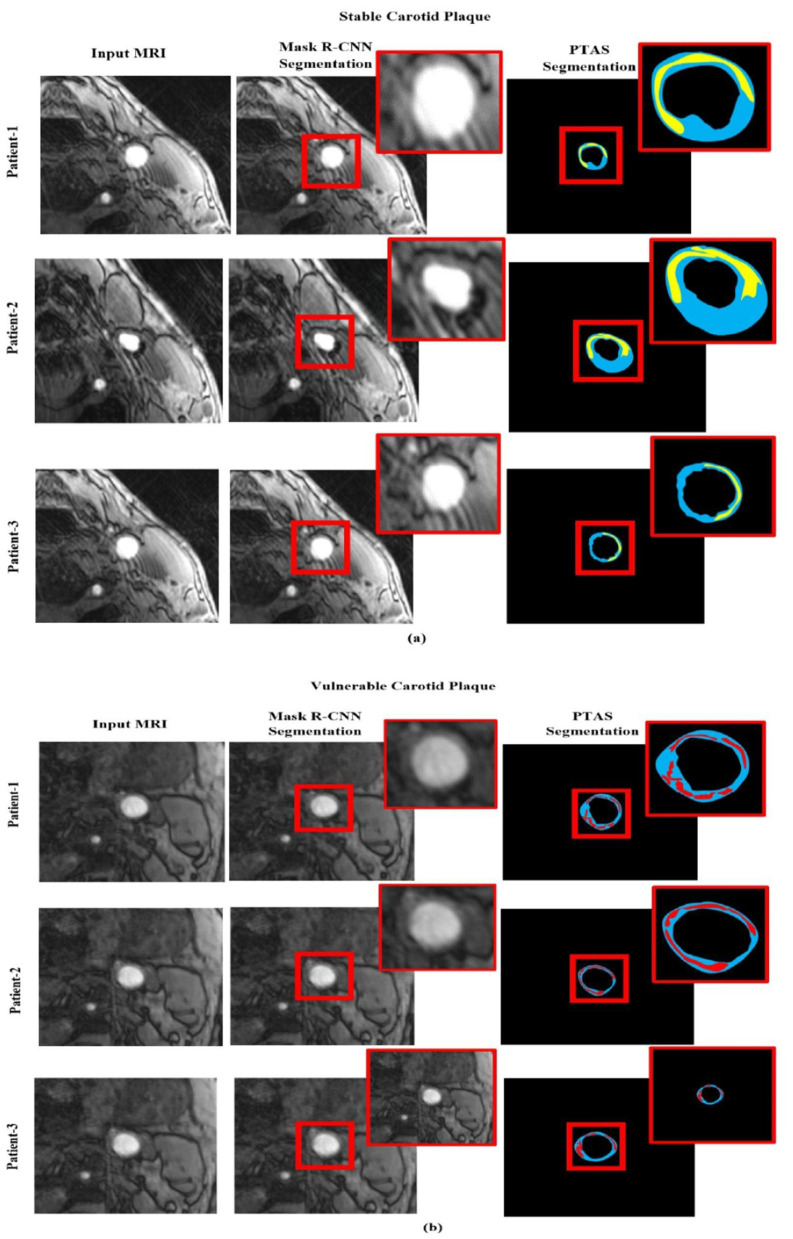
Segmented visualization results using PTAS and Mask R-CNN for two categories: **(a)** stable carotid plaque and **(b)** vulnerable carotid plaque. In the visualizations, the blue region indicates the carotid artery wall, the yellow region represents stable carotid plaque, and the red region denotes vulnerable plaque.

We assess the effectiveness of our Mask R-CNN model using the actual clinical patient data set to determine the method's capacity for segmentation. The testing employs the same evaluation criteria and does not require any fine-tuning. We randomly selected three patients from each class, their magnetic resonance is fed to the model, and their corresponding segmentation images using PTAS and Mask R-CNN for both categories are shown in Figure 8. It provides detailed visual results of our proposed approach for better interpretation. Our approach achieves a much better segmentation performance in contrast to manual segmentation. In Figure 8 blue area is the carotid artery wall, the yellow area is stable carotid plaque, and the red area is vulnerable plaque. Different degrees of red and yellow determine the vulnerability or stability of plaque, respectively.

Whereby Figure 8a is the stable plaque (the blue area is the carotid artery wall, and the yellow area is stable carotid plaque), and Figure 8b represents the images for vulnerable carotid plaque (the blue area is the carotid artery wall, and the red area is vulnerable plaque). In comparison, the first column represents in original magnetic resonance images, while second column are the segmented images using PTAS and third column are the images segmented by using the Mask R-CNN.

### 4.2 Experimental results for plaque classification

**Figure 9:**
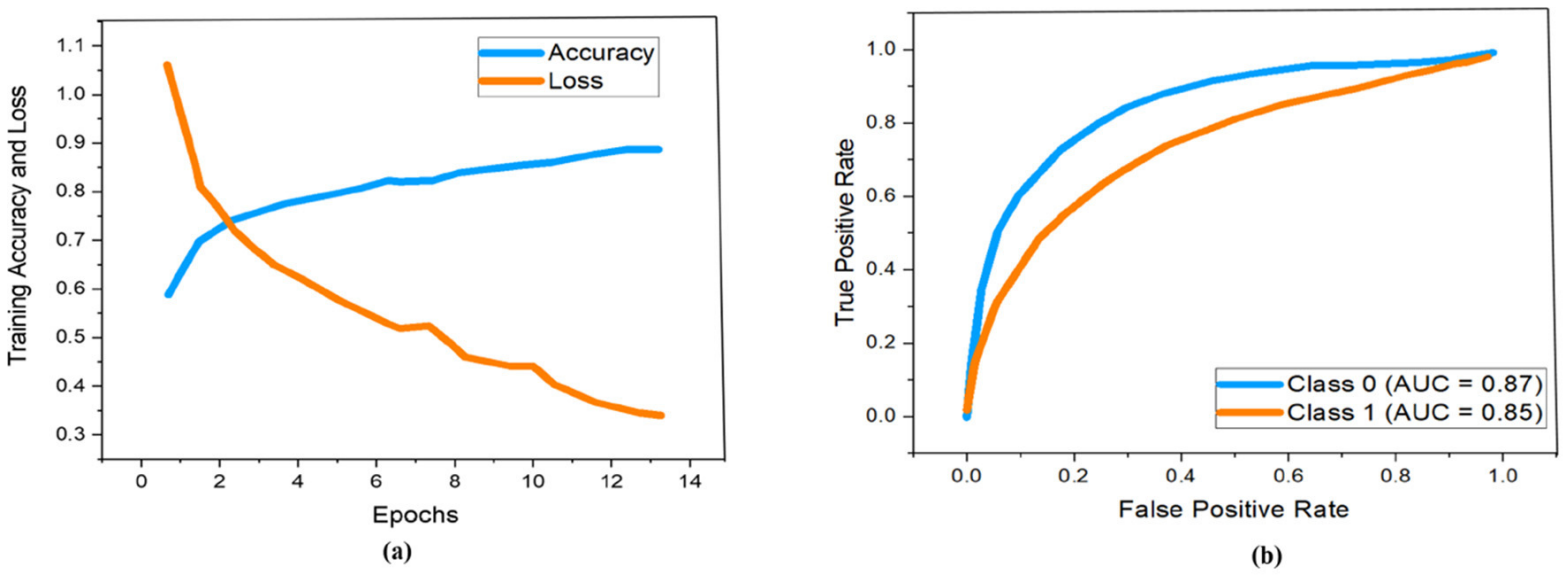
Performance measure using 13-layered CNN according to our dataset. Whereby **(a, b)** represent the training accuracy and loss and ROC curves respectively.

**Figure 10:**
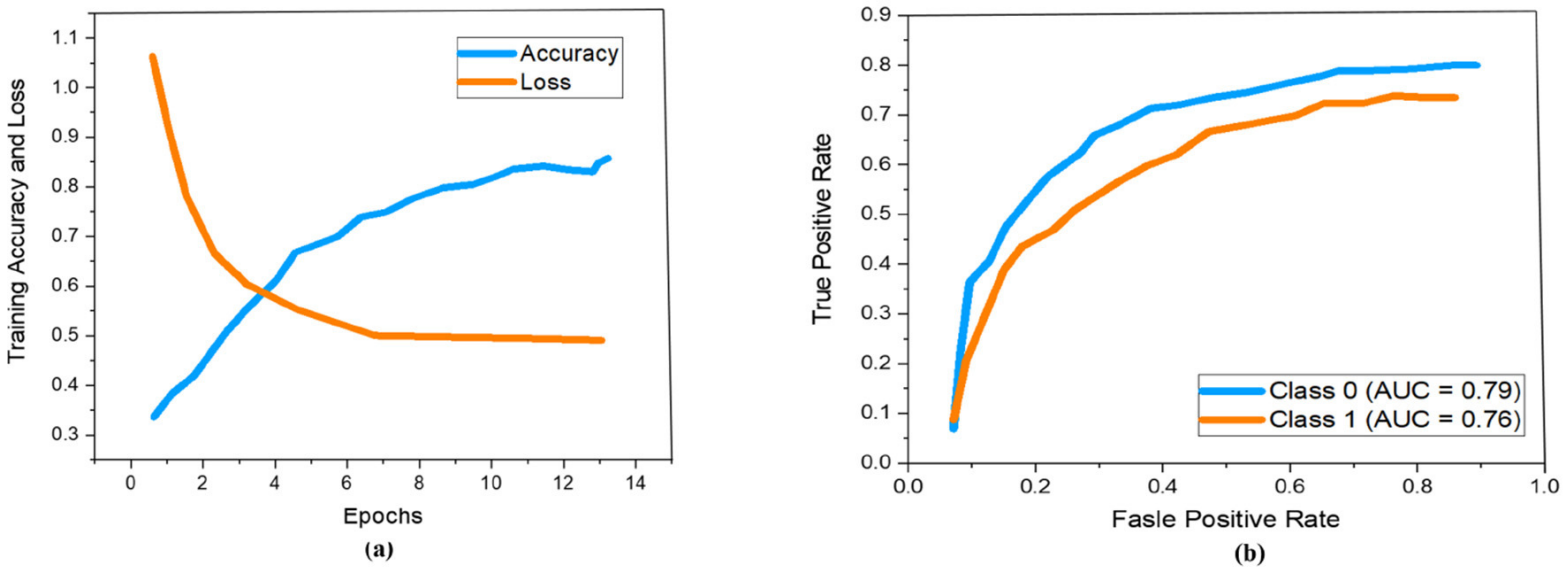
Performance measure using Inception V3 according to our dataset. Whereby **(a, b)** represent the training accuracy and loss and ROC curves respectively.

As demonstrated previously in Figure 5, we used a 13-layered CNN to build the DL architecture, with the final layer serving as the softmax layer. For the purpose of assessing the risk of stroke, the output layer gives us a binary assessment showing that the estimated risk belongs to either the vulnerable or stable carotid plaque. The K10 protocol, which uses 13-layered CNN and MEDCALC 17.0, has the best accuracy and AUC, 88.38% and 0.87 (*p*-value 0.0001), respectively. According to our dataset, Figure 9a displays the training accuracy and loss using a 13-layered CNN. Similarly, by using Inception v3, we achieved 84.21% accuracy in categorizing carotid plaque into two classes as shown Figure 10a shows the training accuracy and loss by using Inception V3.

In the ROC curve, an AUC value close to 1 indicates that the classifier is highly effective at differentiating between positive and negative class points (32). As shown in Figure 9b, the AUC scores for class 0 and class 1 are 0.87 and 0.85, respectively. In comparison, Figure 10b displays AUC values of 0.79 for class 0 and 0.76 for class 1, which are lower than those achieved by the 13-layer CNN. Thus, the 13-layer CNN demonstrated superior performance in classifying magnetic imaging modalities for stroke risk assessment.

**Figure 11:**
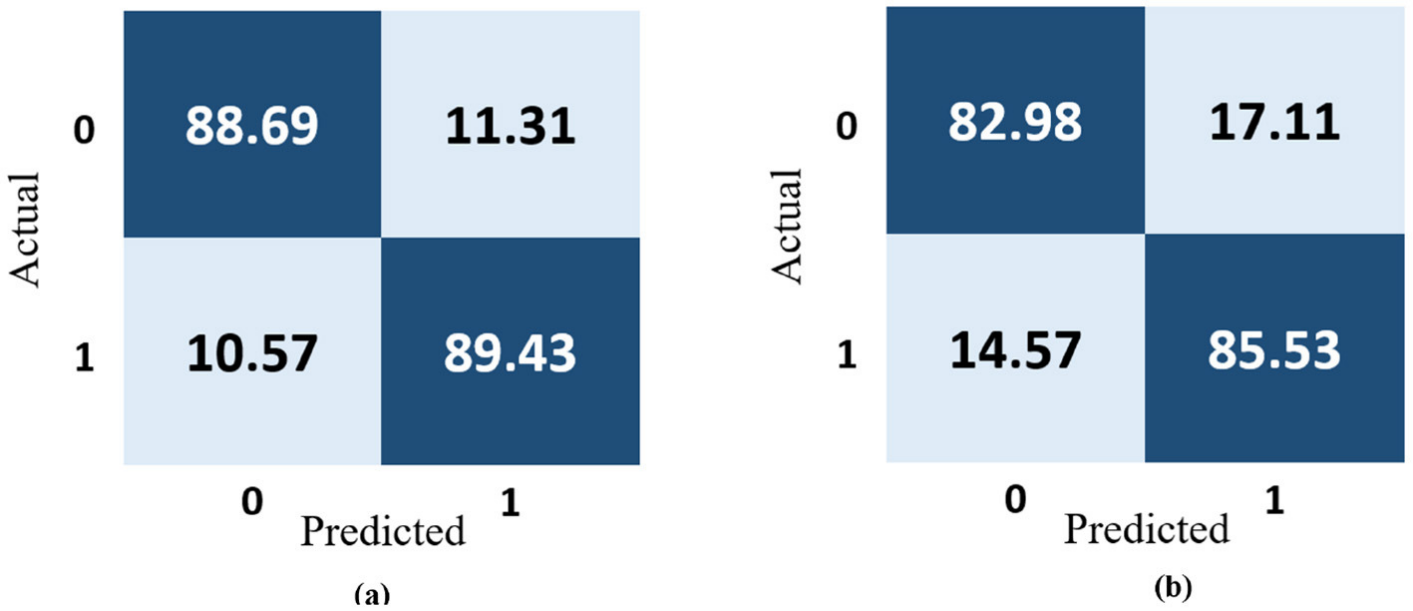
Confusion matrix representation of the performance of the **(a)** 13-layered CNN model and **(b)** Inception V3.

The most popular method for representing experimentally acquired statistical data visually is confusion matrices, which are also used to solve classification problems in deep learning and machine learning methods. Figure 11a shows how well the 13-layered CNN model performed, while Figure 11b shows how well the Inception V3 classified magnetic resonance images into a susceptible and stable carotid plaque for the assessment of stroke risk level using the 22 confusion matrix.

**Figure 12:**
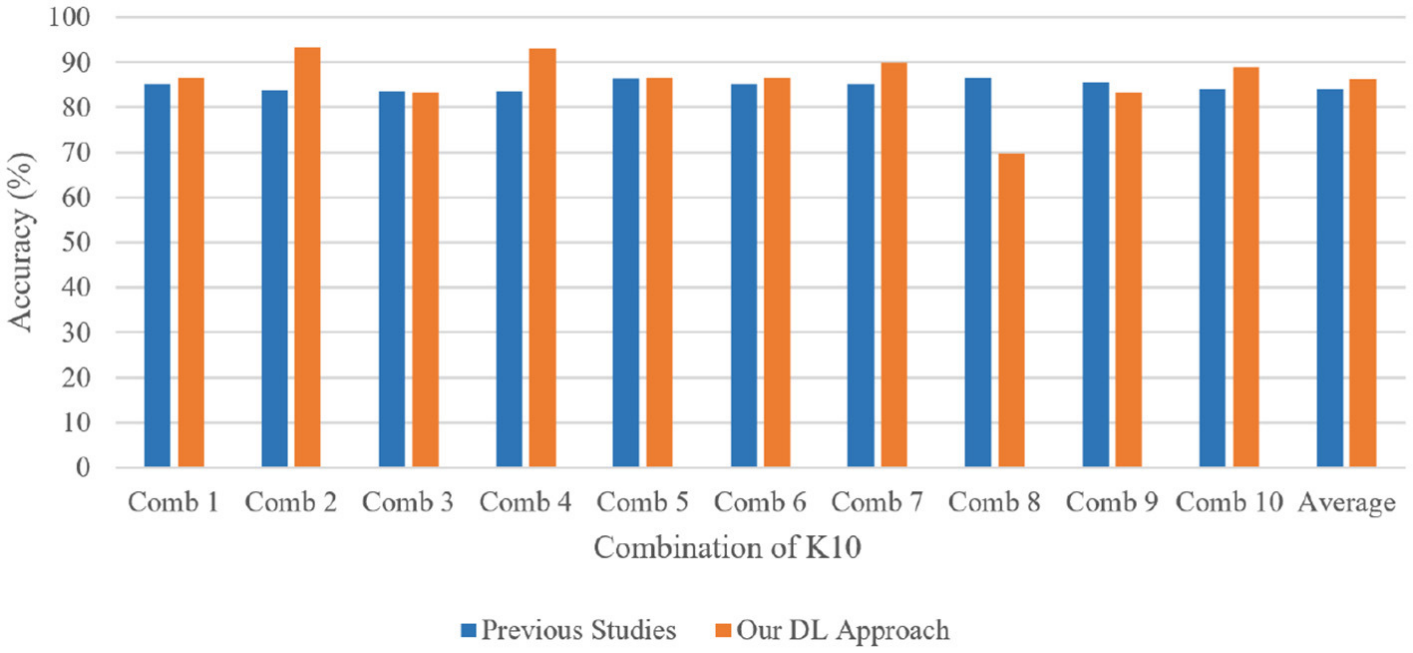
Performance comparison of our proposed 13-layered CNN system with the prior studies using the combination of K10 protocols for identical plaque data.

The 13-layered CNN model classifies 88.69% of the data as True Positive (TP), which indicates that 88.69% falls into this class, as shown in Figure 11a. The 13-layered CNN model incorrectly predicted the positivity of 11.31% of the data, the negativity of 10.57% of the data, and the negativity of 89.43% of the data, which were both negative. Similar to Figure 11b, Inception V3 assigns 82.98% of the data to the True Positive (TP) classification, indicating that 82.98% falls under this category. The Inception V3 incorrectly forecasted 17.11% of the data as positive, 14.57% as negative, and 85.53% as negative of the genuinely negative data; only 14.57% were also predicted to be negative. We calculate each combination's real-time accuracy at the end of the 500th step value to better understand the model performance. We also contrasted the performance of our suggested 13-layered CNN model with that of earlier studies that used the same plaque data (33, 34). The comparative accuracy of previous studies with our proposed approach are displayed in Figure 12 using the K10 cross-validation process.

### 4.3 Comparison with other methods

Our proposed method facilitates the early detection of plaques, with magnetic resonance imaging (MRI) proving more effective than ultrasound for assessing plaque composition and mobility. Integrating both imaging techniques enhances the diagnosis and stability evaluation of carotid atherosclerotic plaques. This approach is crucial for guiding clinical treatment strategies and offers a non-invasive, reliable evaluation method for clinical drug interventions. Additionally, we have compared the performance of our proposed framework with recent state-of-the-art methods published in 2024, 2023, and 2022, as detailed in Table 5.

**Table 5:** Comparative accuracy of the proposed approach with previous studies.

**Reference Study**	**Approach**	**Accuracy**
Jamthikar et al. (35)	Deep learning models	83.98%
Zhang et al. (36)	Deep learning models	80.6%
Li et al. (37)	Two-stage neural network for multi-Weighted MRI	78.0%
Zhou et al. (38)	Image reconstruction-based self-supervised learning (ultrasound)	84.2%
Jain et al. (39)	Deep learning for carotid artery plaque segmentation	85.3%
**Our proposed framework**	**Pre-trained Models (Mask R-CNN + CNN + Inception V3)**	**86.17%**

### 4.4 Ablation analysis

To evaluate the contributions of various model components, we conducted a series of ablation studies to assess the impact of different architectures, loss functions, and learning rate configurations on the overall performance of plaque segmentation and classification. The details are shown in Table 6.

**Table 6:** Ablation study results for plaque segmentation and classification.

**Model variant**	**Architecture**	**Loss function**	**Learning rate**	**Accuracy (%)**	**AUC**
Baseline model	13-layered CNN	Cross-entropy loss	10^−3^	88.38	0.87
Variant 1	Inception V3	Cross-entropy loss	10^−3^	84.21	0.79
Variant 2	13-layered CNN	Dice loss	10^−3^	87.12	0.84
Variant 3	13-layered CNN	Focal loss	10^−3^	88.04	0.85
Variant 4	Mask R-CNN	Cross-entropy loss	10^−3^	85.26	0.82
Variant 5	13-layered CNN	Cross-entropy loss	10^−4^	86.45	0.83
Variant 6	13-layered CNN + Inception V3 (Ensemble)	Cross-entropy loss	10^−3^	**89.15**	0.89

The baseline model, which utilizes a 13-layered CNN with Cross-Entropy Loss and a learning rate of 10^−3^, achieved an accuracy of 88.38% and an AUC of 0.87. We then explored alternative configurations: Variant 1, which replaced the 13-layered CNN with Inception V3, achieved a slightly lower accuracy of 84.21% and AUC of 0.79, indicating that the 13-layered CNN outperforms Inception V3 for this task. In Variant 2, where Dice Loss was applied instead of Cross-Entropy Loss, we observed a slight decrease in performance (accuracy: 87.12%, AUC: 0.84), suggesting that while Dice Loss is effective for segmentation tasks, it is not optimal for classification in this context. Variant 3, using Focal Loss, resulted in improved classification accuracy (88.04%, AUC: 0.85), demonstrating that this loss function can help improve the model's focus on harder-to-classify examples. We also evaluated the Mask R-CNN architecture (Variant 4), which led to a lower performance (accuracy: 85.26%, AUC: 0.82), highlighting that segmentation-specific models may not always translate into better classification outcomes. In Variant 5, we reduced the learning rate to 10^−4^, which resulted in a slight drop in performance (accuracy: 86.45%, AUC: 0.83), suggesting that the learning rate plays a significant role in model convergence. Finally, Variant 6, which combined 13-layered CNN and Inception V3 in an ensemble approach, achieved the highest performance with an accuracy of 89.15% and an AUC of 0.89, demonstrating the benefits of integrating multiple architectures for improved classification. These results collectively highlight the importance of model selection, loss function, and hyperparameter tuning in optimizing performance for plaque detection and risk assessment.

## 5 Discussion

Carotid plaques frequently have been found in patients who suffer from a stroke. Since medical therapy advancements over the past 20 years have decreased the risk of stroke, there is growing interest in finding markers of plaque vulnerability to help identify high-risk individuals. However, drawing firm conclusions on the utility of magnetic resonance imaging carotid plaque characterization is difficult because magnetic resonance images of plaque composition are a relatively new method, and individual studies have often been small. In this study, magnetic resonance imaging approaches with the ability to forecast stroke risk due to carotid atherosclerosis were reported. The methods were arranged in descending order, from those that could be used in clinical settings to those that required the most technological advancement. In this study, we manually segmented the plaque in the carotid arteries using Plaque Texture Analysis Software (PTAS) and automatically segmented the plaque in magnetic resonance images using Mask R-CNN, both of which produced statistically significant results. In addition, we used a 13-layer traditional convolution neural network and Inception V3 to identify high-risk carotid plaque for stroke risk assessment using magnetic resonance images by categorizing carotid plaque into two classes, vulnerable and stable carotid plaque for stroke risk assessment.

## 6 Conclusion

Our study finds that the 13-layer CNN approach stands out as the most effective method for assessing carotid risk. This framework includes two main components: first, automatically segmenting plaque regions from magnetic resonance images, and second, classifying these images into two categories to evaluate stroke risk based on carotid plaque. The 13-layer CNN model we trained achieved an impressive accuracy of 86.17% in classifying magnetic images. We assessed its performance using accuracy and loss graphs, as well as AUC curves, and found that it significantly outperformed previous methods in categorizing carotid plaques for stroke risk assessment. Moreover, we compared our model with earlier studies that used the same plaque data. Our approach addresses potential issues with image quality and individual interpretation, ensuring more reliable diagnoses. The results demonstrate that our method is highly effective for classifying magnetic imaging data to assess stroke risk. Looking ahead, incorporating extreme learning techniques might offer even more advanced solutions for plaque classification.

## Data Availability

The raw data supporting the conclusions of this article will be made available by the authors, without undue reservation.
